# The Need to Start at the Bench

**DOI:** 10.5005/jp-journals-10071-23172

**Published:** 2019-06

**Authors:** Sriram Sampath, Atul P Kulkarni

**Affiliations:** 1 Department of Critical Care Medicine, St John's Medical College Hospital, Bengaluru, Karnataka, India; 2 Division of Critical Care Medicine, Department of Anaesthesiology, Critical Care and Pain, Tata Memorial Hospital, Homi Bhabha National Institute, Mumbai, Maharashtra, India

## Abstract

**How to cite this article:** Sampath S, Kulkarni AP. The Need to Start at the Bench. Indian J Crit Care Med 2019;23(6):245.

It doesn't matter how beautiful your theory is, it doesn't matter how smart you are. If it doesn't agree with experiment, it's wrong.

—Richard P Feynman

In this issue we start a new feature, which includes articles on basic science and experimental research. Ronen and colleagues report the result of a biomechanical study comparing the Ciaglia Blue Rhino® technique (serial dilator) and Portex® technique (using Grigg's guidewire dilator technique) in pigs.^1^ They found that the force required to insert the needle at the beginning, into the trachea and for the final stage of dilatation of trachea using the final dilator with the Ciaglia technique (as compared to the Guide wire dilating forceps) was much greater. Force required to insert the tracheostomy tube was also higher with the Ciaglia kit. The shape of the tracheal mucosal openings and sizes were similar with both the techniques. Lastly the total energy expenditure using the Ciaglia technique was nearly 1.5 times greater than the Girggs technique.

There are several limitations to this study. The force in this experiment was measured using a strain gauge, however, more sophisticated methods of measuring deformation, acceleration, friction, puncture, strength are now available. The force was not differentiated from force along the axis and friction was not measured. While performing an experiment of this type a rigid frame is required, the authors used hands to hold the Griggs forceps. It is therefore difficult to differentiate between the force that may have been applied by hand.

Lastly, but no the least, there was no *in vitro* assessment of damage and trauma to tissue. Also since a bronchoscope was not used, mucosal damage and bleeding risk during the experiment could not be assessed, thus making the *in vitro* assessment suboptimal.

Where does that leave us then? This simple biomechanical experimental study can direct us toward the first step toward deciphering the causes of various complications seen with these techniques. We would caution the readers not to assume that this bench experiment meets the standards needed to guide or alter clinical practice. Though it is a good beginning, many more steps need to be taken before this experiment can translate into clinical practice.
